# Age-dependent effects of moderate differences in environmental predictability forecasted by climate change, experimental evidence from a short-lived lizard (*Zootoca vivipara*)

**DOI:** 10.1038/s41598-019-51955-7

**Published:** 2019-10-29

**Authors:** G. Masó, J. Kaufmann, H. Clavero, P. S. Fitze

**Affiliations:** 1 Department of Biodiversity and Ecologic Restoration, Instituto Pirenaico de Ecología (IPE-CSIC), Avda. Nuestra Señora de la Victoria 16, 22700 Jaca, Spain; 2 School of Biological, Earth & Environmental Sciences, University College Cork, Cork, Republic of Ireland; 3 IUCN-Centre for Mediterranean Cooperation, c/Marie Curie, 22, Edif. Habitec, 29590 Campanillas, Malaga Spain; 4 Department of Ecology and Evolution, University of Lausanne, Biophore, 1015 Lausanne Switzerland; 5 Department of Biodiversity and Evolutionary Biology, Museo Nacional de Ciencias Naturales (MNCN-CSIC), C/José Gutiérrez Abascal 2, 28006 Madrid, Spain

**Keywords:** Projection and prediction, Climate-change ecology, Evolutionary ecology, Theoretical ecology, Population dynamics

## Abstract

Whether and how differences in environmental predictability affect life-history traits is controversial and may depend on mean environmental conditions. Solid evidence for effects of environmental predictability are lacking and thus, the consequences of the currently observed and forecasted climate-change induced reduction of precipitation predictability are largely unknown. Here we experimentally tested whether and how changes in the predictability of precipitation affect growth, reproduction, and survival of common lizard *Zootoca vivipara*. Precipitation predictability affected all three age classes. While adults were able to compensate the treatment effects, yearlings and juvenile females were not able to compensate negative effects of less predictable precipitation on growth and body condition, respectively. Differences among the age-classes’ response reflect differences (among age-classes) in the sensitivity to environmental predictability. Moreover, effects of environmental predictability depended on mean environmental conditions. This indicates that integrating differences in environmental sensitivity, and changes in averages and the predictability of climatic variables will be key to understand whether species are able to cope with the current climatic change.

## Introduction

It is well established that changes in environmental conditions (e.g. climatic conditions) can affect individual performance (e.g. survival, growth rate, and reproduction), life-history strategies and population dynamics^1^. Understanding how organisms cope with and adapt to changes in their environment is thus central for conservation, evolution, and ecology^2^. While it is widely accepted that changes in average environmental conditions affect species’ and individual responses^3^, less evidence exists for effects of environmental predictability (i.e., environmental fluctuations through time^4^) and it is generally thought that less predictable environments negatively affect life history traits^5–7^ and thereby population dynamics^8^.

However, species may behaviourally adapt to such changes in environmental predictability by adapting their life-history strategy^9^, thereby counterbalancing potential negative effects. While a recent review suggests that the effect of environmental variance may depend on mean environmental conditions^10^, no evidence exists that the effects of environmental predictability depends on mean environmental conditions. Moreover, environmental predictability may differentially affect the stages of stage-structured populations^11,12^, through its effect on inter-stage competition^13^, or due to inter-stage differences in the sensitivity to environmental predictability^14^. Therefore, differences may exist in population dynamics, but not necessarily in overall population densities^13^. Consequently, it is far from being clear that less predictable environmental conditions will be consistently negative.

Previous studies predicting negative effects of environmental predictability were mainly theoretical^5,15,16^ or generated extreme events, such as severe droughts^17^. Thus, studies are needed that experimentally test the effects of differences in environmental predictability on life-history strategies and life-history traits, since only those will be able to provide robust evidence for or against these claims^2^.

Robust evidence from experimental work is especially important, since most climate change scenarios predict a long-term increase of rainfall variability, as well as an increase in the frequency of extreme rainfall events^18^. These changes will lead to lower predictability of precipitation and to lower predictability of resources^19^. Precipitation importantly influences habitat, air and soil humidity and thereby entire food webs and their diversity^20^. Precipitation is a vital resource that influences physiology, behavior, life history traits such as body size, body condition, reproduction and survival^21^, and that may reinforce differences among age classes and sexes^22^. Nevertheless, experimental evidence for the importance of precipitation predictability is scarce and limited to studies generating extreme events^17^.

To this end, we experimentally tested in age-structured populations whether and how differences in precipitation predictability affect life-history strategies and life history traits of different age-classes (adults, yearlings, juveniles) of the European common lizard (*Zootoca vivipara*; Lichtenstein, 1823). *Zootoca vivipara* exhibits high dependency on water^22–24^ and water availability constrains its life history traits, e.g., its growth and reproduction^11,25^. Its hydric balance is mainly controlled by environmental factors and behavioural regulation^23^. Here, we experimentally manipulated the predictability of precipitation in 12 semi-natural European common lizard populations, while holding the amount of precipitation constant. The predictability of precipitation was manipulated, given the observed and predicted changes in rainfall patterns^18^ and given the European common lizard’s high dependency on water^11,22–24^. Populations were maintained during one year and the experiment repeated in three subsequent years, to test for the generality of the responses and whether differences in mean climatic conditions may affect differences induced by precipitation predictability.

According to theoretic models^16^, we predicted (1) significant negative effects of less predictable precipitation on body size, body condition, survival, and reproductive traits, such as laying date, litter size, offspring traits and early offspring survival. We also tested (2) whether the effect of precipitation predictability may depend on mean climatic conditions. Because different age-classes generally exhibit different competitive abilities and different investment strategies, potentially leading to intense inter-class competition^11,12,22^, we predicted (3) that effects of precipitation predictability may first manifest in the competitively inferior age-classes, namely in juveniles and yearlings. Alternatively, we predicted (4) that age-class-dependent effects of precipitation predictability may reflect age-dependent sensitivity to precipitation predictability.

## Results

### Climatic differences among years and seasons

Air temperatures significantly differed between periods (*F*_2*,941*_ = 512.450, *P* < 0.001) and no significant differences existed in average daily precipitation (*χ*^2^_2_ = 0.291, *P* = 0.864). Between release and August, average daily air temperature was 19.79 °C ± 0.31 SE, between August and September it was 17.04 °C ± 0.26 SE, and during the rest of the year it averaged 9.36 °C ± 0.22 SE (all pairwise contrasts: *P* ≤ 0.001). The natural average daily precipitation was 2.77 mm ± 0.25 SE.

There was a significant interaction between period (summer: release to August, autumn: August to September) and year in average daily temperatures (interaction: *F*_2*,19*2_ = 38.826, *P* < 0.001, Fig. S1 Supporting Information). In 2013, the temperature decline between summer and autumn was steepest (contrast: *F*_1,2*57*_ = 145.178, *P* < 0.001), shallower in 2012 (*F*_*1,257*_ = 23.170, *P* < 0.001; slope difference 2012 vs 2013: *F*_*1,159*_ = 5.985, *P* = 0.016), and no significant change existed in 2014 (*F*_*1,257*_ = 0.465, *P* = 0.496, Fig. S1 Supporting Information). In summer average daily temperatures were significantly lower in 2014 compared to 2012 and 2013 (all *P* < 0.001), while no significant differences existed between 2012 and 2013 (*P* = 0.802). In autumn daily temperatures were significantly lower in 2013 than in 2014, and they were intermediate in 2015. There was no significant interaction between period and year in daily precipitation (*χ*^*2*^_*2*_ = 0.261, *P* = 0.877), nor significant differences among periods (*χ*^*2*^_*1*_ = 0.066, *P* = 0.797). Moreover, there were no significant differences between periods and years in variances of daily precipitation and temperature (*F*_*5,19*_ = 0.665, *P* = 0.537, *F*_*5,19*_ = 7.75, *P* = 0.944).

In Spring there was a significant interaction between year and month in average daily temperatures (*F*_4,2*67*_ = 5.391, *P* < 0.001; Fig. S2 Supporting Information) and in temperature variance (*F*_4*,18*_ = 3.362, *P* = 0. 032; Fig. S3 Supporting Information), but neither in daily precipitation (*χ*^2^_*4*_ = 4.372, *P* = 0.358), nor in precipitation variance (*χ*^*2*^_*4*_ = 3.051, *P* = 0.549). No significant difference among years existed in average monthly temperatures in March (all post-hoc contrasts: *P* > 0.05), while average April temperatures in 2013 were lower than in 2014 (*P* = 0.015) and intermediate in 2015 (Fig. S2 Supporting Information). In May 2015, temperatures were significantly higher than in the other years, in May 2014 they were intermediate and in May 2013 they were lowest (all differences: *P* < 0.001; Fig. S2 Supporting Information). No significant pairwise contrasts existed among and within years in temperature variance in March, April, and May (all *P* > 0.05; Fig. S3 Supporting Information), showing that small within-year differences caused the significant interaction.

### Treatment effects on survival, growth, and body condition

**Figure 1:**
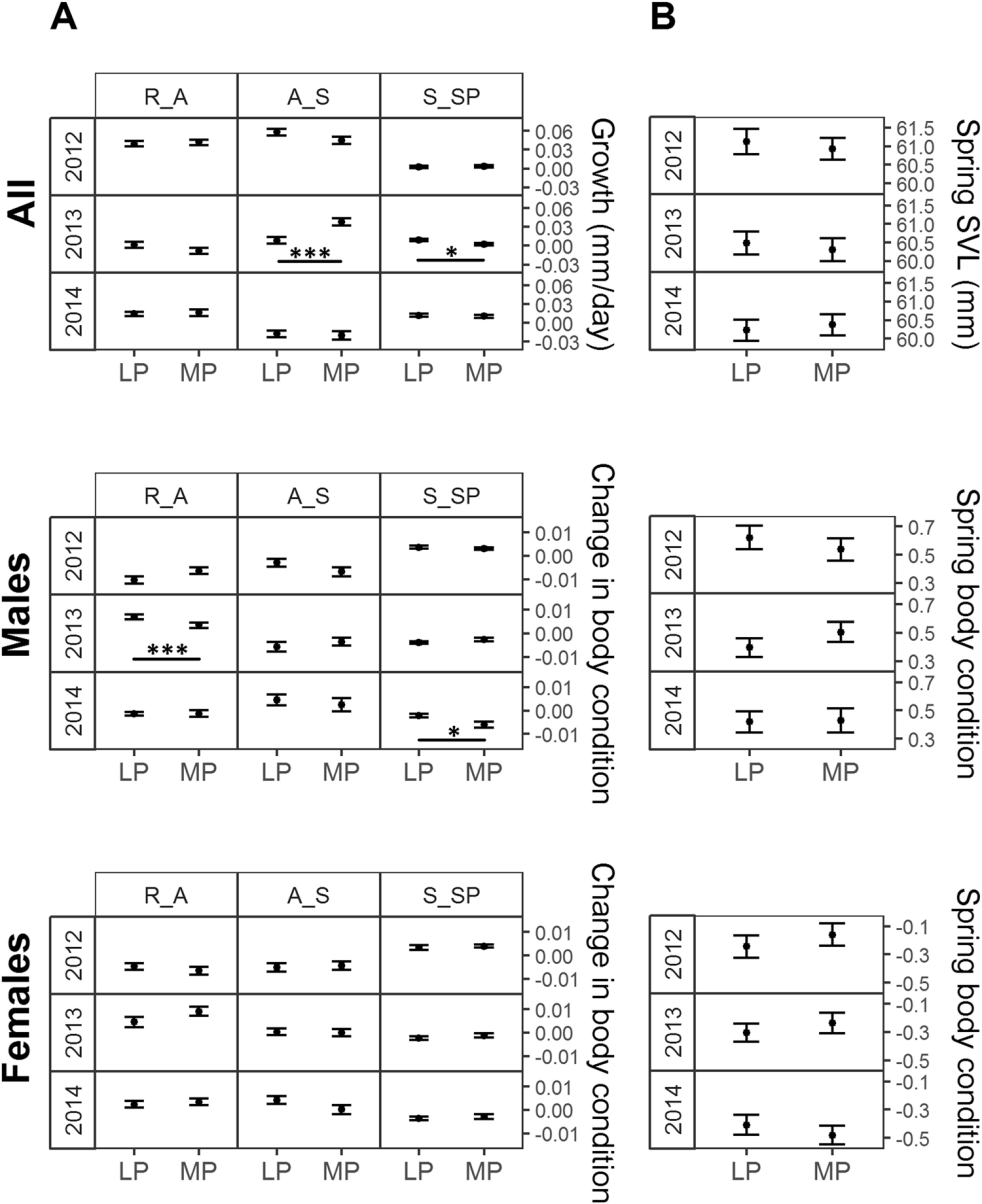
Treatment effects on adult body size (above) and adult body condition (below). In (**A**), changes in SVL and body condition are shown and in (**B**), spring SVL and spring body condition. Predicted means ± se per treatment, growth period, year and sex (in body condition) are shown in (**A**) (LP: less predictable; MP: more predictable; R_A: Release to August; A_S: August to September; S_SP: September to spring) and predicted means ± se per treatment, year, and sex in (**B**). Horizontal lines indicate significant post-hoc contrasts: *P < 0.05; **P < 0.01; ***P < 0.001.

There was no significant treatment effect on survival in any of the age classes (Table 1). In adults, growth was affected by a significant interaction between treatment, year and period (*χ*^2^_4_ = 21.448; *P* < 0.001; Fig. 1A). Post-hoc analyses showed significant differences among environmental predictability treatments in 2013. From August to September 2013 growth was significantly faster in the more predictable treatment (*P* < 0.001) and from September to spring it was significantly slower (*P* = 0.012), the latter effect compensating the former effect. As a result, no significant differences existed between treatments in spring SVL (*χ*^*2*^_1_ = 0.158; *P* = 0.691; Fig. 1B). In adult lizards, the change in body condition was affected by a significant interaction between treatment, sex, year and period (*χ*^2^_4_ = 13.917; *P* = 0.007; Fig. 1A). Post-hoc analyses showed that in 2013 and 2014 significant differences among treatment levels existed in males, while no significant differences existed in females (Fig. 1A). The change in male body condition was bigger in the less predictable treatment from release to August 2013 and from September to spring 2014. However, no treatment effects existed in spring body condition (*χ*^2^_1_ = 0.231; *P* = 0.631; Fig. 1B), showing that in 2013, this difference was compensated by small differences in subsequent periods and that in 2014, the treatment effect compensated previous small differences.

**Table 1:** Summary of treatment effects on survival, body size, body condition, growth, and change in body condition.

Response variable	Treatment effects	df	Adults			Yearlings			Juveniles		
			N	χ2	P	N	χ2	P	N	χ2	P
survival	treatment	1	888	—	—	240	—	—	788	—	—
growth	treatment × period	2	1799	—	—	486	19.591	<0.001	124	—	—
	treatment × year × period	4	1799	21.448	<0.001	486	—	—	124	—	—
change in body condition	treatment × sex × year × period	4	1777	13.917	<0.001	478	—	—	124	—	—
spring body size	treatment	1	623	—	—	156	4.799	0.028	57	—	—
spring body condition	treatment × sex	1	595	—	—	146	—	—	57	6.679	<0.001

Treatment effects (environmental predictability) and interactions including treatment are given. Significant P-values or ‘—‘ for non-significant parameters are given. Minimum adequate models and test statistics are shown in Supporting Information Tables S2–S4.

**Figure 2:**
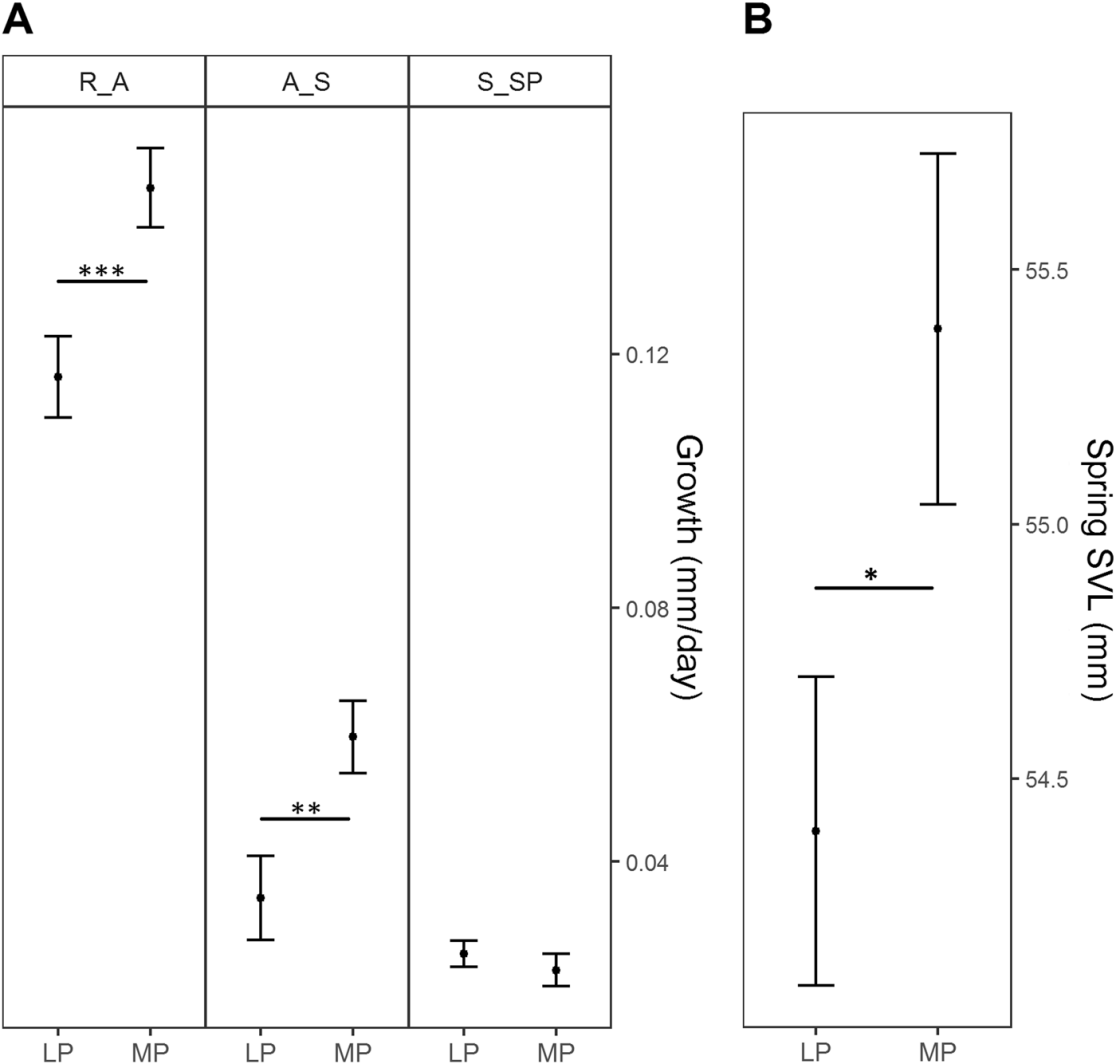
Treatment effects on yearling growth (**A**) and spring SVL (**B**) of yearlings. For growth, predicted means ± se per treatment and growth period (LP: less predictable; MP: more predictable; growth period: R_A: Release to August; A_S: August to September; S_SP: September to spring) are shown and for spring SVL, predicted means ± se per treatment. Horizontal lines indicate significant post-hoc contrasts: *P < 0.05; **P < 0.01; ***P < 0.001.

In yearlings, growth was affected by a significant interaction between predictability treatment and period (*χ*^2^_2_ = 19.591; *P* < 0.001; Fig. 2A). In the more predictable treatment, yearlings grew significantly faster before hibernation (i.e. from release to august and from august to September) than in the less predictable treatment and no significant differences existed between September and spring. Consequently, spring SVL was significantly longer in the more predictable treatment (*χ*^2^_1_ = 4.799; *P* = 0.028; Fig. 2B). Treatment did not significantly affect the change in body condition or spring body condition of yearlings (*P* > 0.05; Table 1).

In juveniles, treatment did not significantly affect growth nor spring SVL (Table 1). Spring body condition was significantly affected by an interaction between predictability treatment and sex (*χ*^2^_1_ = 6.679; *P* = 0.009; Fig. S4B Supporting Information). Post-hoc analyses showed that in the less predictable treatment body condition of males was higher than in females, while no significant sex-differences existed in the more predictable treatment (Fig. S4B Supporting Information). There was no significant interaction between treatment and sex on the change in body condition (*χ*^2^_1_ = 0.526; *P* = 0.468; Fig. S4A Supporting Information), but graphical representation of the interaction unraveled trends that are congruent with the significant treatment × sex interaction on spring body condition (Fig. S4A Supporting Information).

### Treatment effects on reproduction

**Figure 3:**
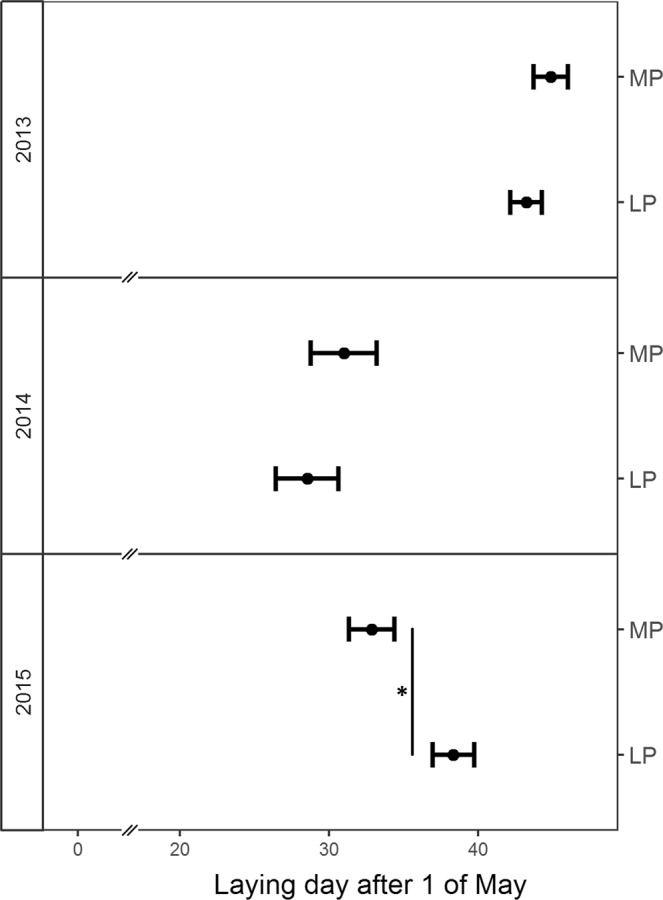
Treatment effect on laying date (1st of May corresponds to day 0). Shown are predicted means ± se per treatment (LP: less predictable; MP: more predictable) and the year the experiment started (note: in the 2012 experiment, laying date was measured in spring 2013, in the 2013 experiment it was measured in spring 2014, etc). Vertical lines indicate significant post-hoc contrasts: *P < 0.05; **P < 0.01; ***P < 0.001.

Laying date was affected by a significant interaction between treatment and year (*χ*^2^_2_ = 8.887; *P* = 0.012; Fig. 3). Post-hoc analyses show that in spring 2015, lizards in the more predictable treatment laid eggs significantly earlier than those exposed to the less predictable treatment. In the other years, no significant treatment effects existed. Treatment did not affect the other reproductive parameters (Table 2).

**Table 2:** Minimum adequate models and test statistics for the analysed reproductive traits are given.

*Parameter*	*df*	Clutch size		Date num		Hatching S.		N° Juveniles		Laying S.		Mat. Invest.	
		*χ2*	*P*	*χ2*	*P*	*χ2*	*P*	*χ2*	*P*	*χ2*	*P*	*χ2*	*P*
Treatment	1	—	—	0.137	0.712	—	—	—	—	—	—	—	—
**Age**	1	**5.577**	**0.018**	**18.757**	<**0.001**	1.772	0.183	—	—	1.772	0.183	**7.086**	**0.008**
**Year**	2	**18.755**	<**0.001**	**88.206**	<**0.001**	**8.477**	**0.014**	**9.510**	**0.009**	**8.477**	**0.014**	—	—
Treatment × Age	1	—	—	—	—	—	—	—	—	—	—	—	—
**Treatment** × **Year**	2	—	—	**8.887**	**0.012**	—	—	—	—	—	—	—	—
**Age** × **Year**	2	—	—	—	—	**6.306**	**0.043**	—	—	**6.306**	**0.043**	—	—
Treatment × Age × Year	2	—	—	—	—	—	—	—	—	—	—	—	—
Sample size (*N*)		373		683		372		375		683		253	

Significant factors and/or interactions are shown in bold.

## Discussion

Climate change research predicts a decrease in the predictability of precipitation^18^, potentially having negative effects on species viability, population growth, and individual performance^5,7^. Most studies on this subject are theoretical^5,16^ or correlate environmental predictability with life-history traits^4,6,7^, and the few experimental studies produced extreme events that severely affected life-history traits^17^. Moreover, while it has been predicted that the effect of environmental variance may depend on mean environmental conditions^10^, no evidence exists that the effects of environmental predictability depends on mean environmental conditions. Thus, robust experimental proof for the effects of environmental predictability are lacking^2^. Here we experimentally test whether life-history strategies and life-history traits are affected by moderate differences in the predictability of precipitation, i.e., without producing extreme events (i.e., droughts or floods), and whether differences in mean climatic conditions alter their effects.

Our results show that reduced precipitation predictability negatively affected growth of yearlings, body condition of juvenile females (Table 1; Figs 2, S4 Supporting Information), and the timing of egg laying (Table 2, Fig. 3) of common lizards. Precipitation treatment significantly affected adult growth rate and body condition of adult males (Fig. 1A), and all effects were compensated over the course of the experiment, leading to no significant differences on adult spring traits (Fig. 1B). These findings are in line with theory suggesting that less predictable environments negatively affect life history traits^5–7^ and especially those of competitively inferior age classes, namely, yearlings and juveniles^12^. The absence of treatment effects on adult spring traits, further points to highly plastic compensatory strategies that mitigate environmentally induced effects on traits relevant to sexual selection^26,27^. Moreover, reduced growth in the less predictable treatment in autumn 2013, is congruent with reduced growth of yearlings, potentially pointing to inter-age class competition^12^. However, absence of treatment effects on juvenile growth are not congruent with the predicted cascading effects and thus, with the prediction that effects of precipitation predictability may first manifest in the competitively inferior age-classes, i.e. juveniles followed by yearlings (prediction 3)^12^. Consequently, it is unlikely that the differential treatment effects observed in the different age classes are the result of treatment-induced differences in inter-age class competition (for further discussion see in Supporting Information Appendix S2).

The inter-annual differences in age-class dependent treatment effects, as well as sex-dependent treatment effects, show that there exist age-class and sex-dependent differences in the sensitivity to differences in precipitation predictability^14^. Different sensitivities may result from differences in water flux^22^ and/or thermoregulatory activity^28^. In *Z. vivipara* water flux positively correlates with growth rates and in summer, gravid females exhibit higher water fluxes than males and non-gravid females, while in autumn no significant differences exist between sexes and age-classes^22^. Differences in water fluxes between sexes and age-classes can thus hardly explain the here observed patterns, while differences in thermoregulatory activity are congruent with the observed effects. If precipitation is falling, common lizards hide and are thus not able to thermoregulate, and differences in thermoregulation directly feed back into growth and timing of egg laying^29^. Moreover, thermoregulatory capacity is known to depend on body size^28^ and on coloration^30^. Larger animals (adults) have higher thermal inertia and can keep heat for longer^28^, thus they might be less affected by precipitation predictability, and similarly, juveniles exhibit darker coloration that leads to faster heating^30^, what may explain the absence of treatment effects on juvenile growth (for further discussion see in Supporting Information Appendix S3).

Theory indicates that reduced predictability negatively affects life history traits^5^, but here, the predictability of precipitation did neither affect survival, nor most of the reproductive traits (Table 1, 3.2). This suggests that reduced precipitation predictability may not necessarily negatively affect life-history traits and since adults compensate treatment-induced effects, it is also unlikely that it affects sexual selection^27^. However, predictability treatment affected spring body size of yearlings, which may lead to a delay in maturation and to differences in clutch size^31^, both potentially affecting population dynamics.

Finally, treatment effects on adults (growth, body condition, laying date) were indeed year-specific (Table 1), while treatment effects on yearlings and juveniles were independent of year. Adult growth was reduced in the less predictable treatment in 2013, when average autumn temperature was lowest (15.312 °C ± 0.400 SE in 2013, 16.988 °C ± 0.564 SE in 2012 and 18.833 °C ± 0.360 in 2014, Fig. S1 Supporting Information), but not in the other years. Laying date was affected by a significant interaction between year and predictability treatment (Fig. 3) and there existed a significant interaction between year and month in average monthly temperatures (Fig. S2 Supporting Information) and no significant interaction between year and month in daily precipitation, nor in precipitation variances. In the 2013 experiment, April temperatures were highest (see temperatures during April 2014, Fig. S2 Supporting Information) and eggs were laid earliest (average date: give data: 30^th^ of May ± 1.49 SE) and in the 2012 experiment, April temperatures were coolest (see temperatures during April 2013, Fig. S2 Supporting Information) and eggs were laid latest (average date: give data: 14^th^ of June ± 0.77 SE, Fig. 3), while in the 2014 experiment average daily temperatures and date of egg laying were intermediate (5^th^ of June ± 1, Figs 3, S2 Supporting Information). Average April temperatures significantly predicted laying date (*F*_*1,1*_ = 157451, *P* = 0.002), while May temperatures did not (*F*_*1,1*_ = 0.501, *P* = 0.61). This points to reproductive activities being induced by April temperature, which is in line with previous findings showing that emergence of *Z. vivipara* females from hibernation mainly depends on maximum daily temperatures^32^.

In the 2014 experiment, females laid their eggs on average 5 days earlier in the more predictable (3^th^ of June ± 1.48) compared to the less predictable treatment (8^th^ of June ± 1.36, Fig. 3), a result that clearly contrasts to the classic tenet, that lower predictability should favour rapid reproduction^15^. However, no significant treatment effects on laying date existed in the 2012 and 2013 experiments (Fig. 3). This suggests that effects on laying date and adult growth, but not those on yearlings and juveniles, might be the consequence of differences in average climatic conditions. The results on adults show for the first time that population responses to differences in environmental predictability depend on mean conditions.

In summary, the results show for the first time that different age classes and sexes exhibit different sensibilities to the predictability of precipitation^14^. However, the simulated moderate differences in precipitation predictability^19^ had no lasting effects on life history traits of adults and juveniles^5–7^ and no effects on parameters being directly related to population growth^8^. This suggests, that moderate changes in the predictability of precipitation, may not necessarily affect population densities and population persistence, which clearly contrasts to earlier studies, where a decrease of precipitation predictability led to severe droughts and associated strong negative effects on life-history traits and reproduction^17^. Consequently, as long as the predicted decrease in rainfall predictability^18^ will not lead to an increase in the magnitude and frequency of extreme events, its effect on populations, species and biodiversity may not necessarily be negative.

## Materials and Methods

### Species description

The European common lizard, *Zootoca vivipara* is a small ground-dwelling ovoviviparous lizard (adult snout-to-vent length (SVL): 45–70 mm). It is distributed across Europe and northern Asia. It inhabits humid habitats, and microhabitat choice is linked to soil humidity^24^. *Zootoca vivipara* has a highly permeable skin, which increases the risk of hydric loss^22^. Environmental factors and behavior, mainly control the hydric balance of this species^23^. Water availability constrains growth and reproduction, litter size, juvenile performance and size at hatching^11,25^. Growth mainly depends on food intake, activity time, ambient and body temperature, and thus on food availability and the time available for thermoregulation^31,33^. In most *Z. vivipara* populations, three age classes can be distinguished based on body size and coloration^12^: juveniles are entire black and yearlings, and adults exhibit grey-brown cryptic dorsal coloration. Growth rate is highest in juveniles, followed by yearlings, and it is lowest in older age-classes^11^. Mortality of juveniles is high (up to 90%) and once survived the first year, life span is 5–6 years in females and 4–5 years in males^11^. Adults are competitively superior over yearlings and juveniles^11,12,34^, due to size-dependent dominance^12^. Prey size consumed by juveniles and adults largely overlap (Pianka’s index 0.62–0.74)^35^. While adults consume the entire prey size range consumed by yearlings and juveniles, juveniles and yearlings cannot consume the bigger prey eaten by adults^35^, because the size of the consumed prey principally depends on the size of their mouth.

### Experimental setup

#### Environmental procedures

To test how differences in environmental predictability affect life-history strategies and reproduction of *Z. vivipara*, we established 12 age-structured lizard populations in enclosures with natural vegetation located at the Research Station ‘El Boalar’ (Jaca, Spain; for details see^11,12^). Environmental predictability was manipulated on the enclosure level over four years (2012–2015). During these four years, average monthly temperatures (from a measurement station located less than 500 meters from the enclosures and shared by AEMET) increased from February (2.533 °C ± 0.736 SE) to July (19.795 °C ± 0.736 SE) and thereafter decreased until February (Fig. S2–1 Supporting Information). There was a significant quadratic relationship between average monthly temperatures and month (month: *F*_*1,42*_ = 18.050, *P* < 0.001; month^2^: *F*_*1,42*_ = 177.740, *P* < 0.001) explaining 80% of the variation, and no significant differences of this relationship existed among years (all *P* > 0.9). In contrast to temperature, no significant relationships existed between the amount of monthly precipitation and month, and all monthly standard errors overlapped (all *P* > 0.8, Fig. S3–1 Supporting Information). Six enclosures were exposed to more and another six to less predictable precipitation, by supplementing precipitation with 4 sprinklers per enclosure, one in each corner, ensuring homogeneous precipitation. In the more predictable treatment, one supplemental precipitation event happened every day at 9.00 am and at 6.00 pm (i.e., 14 supplemental precipitation events per 7 days, each providing the same amount of precipitation). In the less predictable treatment, 14 supplemental precipitation events were randomly distributed among 7 days between 9.00 am and 7.00 pm. The natural and the supplemental precipitation together, correspond to more and less predictable precipitation, which was confirmed by weighted permutation entropy^36^ (for calculation details see Supporting Information Appendix S1). Permutation entropy was larger in the less (0.86) and smaller in the more predictable treatment (0.77), showing that precipitation was less predictable in the less predictable treatment. Consequently, all enclosures obtained the same amount of precipitation, while the predictability significantly differed among precipitation treatments.

#### Animal release and re-capture protocol

Lizards used for this experiment were originally captured from natural populations located in Aragón and Navarra, and they belong to the North-East Spain subclade, B4^37,38^. The capture and handling of lizards were conducted under the license provided by the Gobierno de Aragón (LC/ehv 24/2010/105 and 106) and Gobierno de Navarra. The conducted study complies with current Spanish laws and Association for the Study of Animal Behavior/Animal Behavior Society guidelines for the treatment of animals in behavioral research.

In July of each experimental year, all lizards that have previously been captured in the enclosures were released back to the enclosures. In 2013 and 2014, half of the lizards of each sex and age class were released in the same predictability treatment (but in an unknown enclosure), while the other half was released in the other predictability treatment. In each enclosure, the same number of adults and yearlings, and a similar number of juveniles was released (Table S1). No significant differences existed among treatments in the number (*F*_*1,10*_ = 1.033; *P* = 0.317) and sex-ratio (*F*_*1,10*_ = 0.005; *P* = 0.940) of released juveniles. Lizards were randomly attributed to enclosures and no significant differences existed among treatment levels in SVL, body condition and adult male color morph frequency (all *P* ≥ 0.2). Adult and yearling lizards were released in July, females after parturition, and juveniles two days after hatching. All adults and all yearlings were released in an unfamiliar enclosure, i.e. not in the enclosure where they have been captured previously. Juveniles belonging to the same clutch were released together and not in the enclosure where the mother was recaptured before egg laying. Moreover, to avoid effects of mother-offspring competition^39^, juveniles were released in a different enclosure than their mother. All used lizards were individually marked by toe-clipping. After release, two recapture sessions were conducted, one at the end of August and the other one at the end of September, and each consisted of three consecutive days of capture with equal capture effort across time and enclosures. After measurement, lizards were released in the same enclosure and in the same location where they were recaptured. If a lizard was recaptured on several days within the same recapture session, measures of the first recapture were used for analyses. Lizards hibernated in the enclosures and in spring (from mid-April onwards), females were recaptured weekly and gravidity was determined by means of belly palpation. Gravid females were moved to the laboratory where they were kept in individual terrariums under standardized conditions (for details see^12^), while non-gravid females were released in the exact recapture location directly after recapture. Terraria of gravid females were checked twice a day for laid clutches which were thereafter incubated individually under standardized conditions^12^. After the mating season (approximately end of May), all surviving lizards were recaptured and brought to the laboratory. Thereafter, all individuals were kept in individual terrariums until release following the protocol used by San-Jose *et al*.^12^. Recapture of all surviving individuals was assured by searching a given enclosure for lizards until five days passed since the last lizard has been recaptured in this enclosure. All detected lizards were recaptured.

### Measures and statistical analysis

Before release and at each capture, SVL (accuracy: 1 mm) and body mass (accuracy: 1 mg) were measured. For each gravid female, body mass before and after parturition, laying date, clutch size, number of removed eggs (sterile or containing a dead embryo), and the number of hatchlings were measured. For laying date, the 1st of May corresponds to day 0 (2nd of May to day 1, etc.) in each studied year. From this data the following parameters were derived: annual survival (yes/no), probability of egg laying (yes/no), proportion of alive juveniles (N_hatchlings_/clutch size), and maternal investment, which corresponds to the difference in body mass before laying minus body mass after laying. Body condition corresponds to the residuals of a linear regression of body mass on SVL. Growth rate and change in body condition were calculated as difference in SVL and body condition between captures divided by the number of days passed between captures $(\frac{trai{t}_{(latercapture)}-trai{t}_{(earliercapture)}}{{N}_{daysbetweencaptures}})$. Both variables were calculated for three different periods: from release to August, from August to September and from September to spring. For the latter period, the days spent hibernating were subtracted (1st of November to 1st of March)^12^.

Mixed models^40^ were ran using treatment, sex and year as fixed factors and enclosure as random factor. Year refers to the period from the yearly set-up of the experimental populations in July to recapture and egg laying in the following year. For example, individuals released in 2012, recaptured in 2013, and egg laying of females in 2013, were modelled as belonging to 2012. In the case of repeated measures taken on the same animal, “animal ID” was modelled as random factor. Finally, in models on juveniles, “Mother ID” was modelled as a random factor since individuals belonging to the same clutch are not independent. Because individuals living in the same enclosure are not independent, the enclosure/year combination was modelled as a random factor, when the analysed data included all three years. Linear mixed models with Gaussian error distribution were applied to analyse treatment effects on SVL, body condition and most reproductive traits. Generalized mixed models with binomial error distribution were used to analyse survival, the probability of egg laying and the proportion of alive juveniles. In analyses including data of different periods, period was modelled as fixed factor and its interactions with the other parameters was included.

Natural climatic data (daily precipitation and average daily temperature) was analysed using general linear mixed models with Gaussian error distribution for average daily temperature and with Poisson error and a log link for daily precipitation. To test for differences among periods, factor “period” was included as a fixed factor. To test if variance in climatic parameters was different among month, periods and years, each month was cut into three equal intervals (from 1.-10., from 11.-20., from 21.-30. or -31.) and variance was calculated for each interval.

Model selection started with the full model that included all parameters and all possible interactions, and the minimum adequate model was determined using backward elimination (see Tables 1 and 2, and in supporting information see Tables S2–S4). For all tests, the significance level was set at α = 0.05 (two-tailed test). All model assumptions were tested and if they were not met, transformations were applied. If heteroscedasticity still existed, weighted least square regressions were ran. Overdispersion existed in none of the non-Gaussian models. For significant factors containing more than two levels, post-hoc tukey-tests accounting for multiple testing were run. All analyses were run using R 3.5.1^41^.

## Supplementary information

Supplementary information

## Data Availability

The data associated with this manuscript is available from the public repository DIGITAL.CSIC on http://hdl.handle.net/10261/192533.
