# Supplementary material for: Age-dependent effects of moderate differences in environmental predictability forecasted by climate change, experimental evidence from a short-lived lizard (Zootoca vivipara)
Source: Sci Rep. 2019 Oct 29;9:15546. doi: 10.1038/s41598-019-51955-7 (PMC6820789; doi:10.1038/s41598-019-51955-7)
Supplement: Supplementary file 1 — Supplementary information [file 41598_2019_51955_MOESM1_ESM.docx]

Supplementary information

**Age-dependent effects of moderate differences in environmental predictability forecasted by climate change, experimental evidence from a short-lived lizard (*Zootoca vivipara*).**

G. Masó^1^, J. Kaufmann^2^_,_ H. Clavero^3^ and P.S. Fitze^1,4^

^1^Department of Biodiversity and Ecologic Restoration, Instituto Pirenaico de Ecología (IPE-CSIC), Avda. Nuestra Señora de la Victoria 16, 22700 Jaca, Spain.

^2^School of Biological, Earth & Environmental Sciences, University College Cork, Cork, Republic of Ireland.

^3^Department of Ecology and Evolution, University of Lausanne, Biophore, 1015 Lausanne, Switzerland.

^4^Department of Biodiversity and Evolutionary Biology, Museo Nacional de Ciencias Naturales (MNCN-CSIC), C/José Gutiérrez Abascal 2, 28006 Madrid, Spain.

^*^ Corresponding author: Patrick S. Fitze; e-mail: patrick.fitze@mncn.csic.es

Index of supplementary information

**Appendix S1**. Specific information of the calculation of the weighted permutation entropy.

**Appendix 2**. Detailed explanation of the absence of evidence that treatment affected inter-age class competition.

**Appendix 3**. Detailed explanation of the potential causes of differences between age-classes in the sensitivity to precipitation predictability.

**Table S1**. Number of adults, yearlings, and juveniles released per enclosure and year.

**Table S2, S3 and S4**. Minimum adequate models and test statistics of growth, change in body condition, spring SVL and body condition and survival for adults, yearlings and juvenile lizards.

**Figure S1, S2, S3 and S5**. Plots of the climatic variables.

**Figure S4**. Plot of the treatment effects on the change in body condition and on spring body condition of juveniles.

**Appendix S1**. Calculation of weighted permutation entropy.

Weighted permutation entropy was calculated to quantify the predictability of precipitation in each treatment level. Permutation entropy is a measure of time series complexity that is inversely related to intrinsic predictability. Time series with high permutation entropy have low redundancy and low intrinsic predictability^1^. Weighted permutation entropy was calculated for each environmental predictability treatment and year based on the obtained daily amount of precipitation (natural precipitation plus supplemental precipitation) during the lizards’ active season (from March to October). To calculate the permutation entropy, time series are encoded as permutation patterns (see e.g. Riedl *et al.*)^2^ which reflect the rank order of *n* successive measures (*x_i_*). Weighted permutation entropy is then calculated according to:

$$H_{n}= -\sum_{j=1}^{n!} p_{j}{log}_{2}(p_{j}) /{log}_{2}(n!)$$

where *p_j_* represents the relative frequencies of the observed permutation patterns^3^. The number of successive measures (*n*) included in a permutation pattern depends on the time-series length (for more details see^4^ and the optimal *n* of the sequence used to encode permutation patterns was four.

**Appendix S2**. Absence of evidence that treatment affected inter-age class competition.

If inter-age class competition explains differences between age-classes in the treatment’s effect, we predicted cascading effects and thus, that effects of precipitation predictability may first manifest in the competitively inferior age-classes (prediction 3). Such hierarchic treatment effects, would predict immediate cascading effects on inferior age-classes^5^. For example, if a given treatment induces food shortage, adults may be able to cope with this situation by eating all size classes of prey consumed by inferior age-classes (in *Z. vivipara* adults eat Collembola, prey eaten by juveniles;^5^ and by excluding inferior age-classes from food^6^. As a consequence, treatments may affect traits of inferior age classes, but not those of adults (e.g. San-Jose *et al.* 2016)^5^. Alternatively, adult traits may also be affected by treatment, but less than those of inferior age classes. In this study, the observed differences among age-classes in the treatment’s effect (i.e. between adults and the inferior age classes) were not congruent with predictions from inter-age class competition. First, adult males of the more predictable treatment exhibited a significantly smaller positive change in body condition between release and august 2013 than those of the less predictable treatment (Fig. 3A). This suggests that inter-age class competition may have been higher in the more predictable treatment, which should lead to negative effects on yearlings and/or juveniles. Nevertheless, in the more predictable treatment growth of yearlings and the body condition change of female juveniles was bigger compared to the less predictable treatment (Figs. 4A, 5A) and thus inter-age class competition, e.g. by means of food competition, cannot explain these patterns. Second, in 2014 the body condition change of adult males from September to spring was more negative in the more predictable treatment (Fig. 3A). This suggests that inter-age class competition may have been higher in the more predictable treatment, but yearling traits (growth and change in body condition) were not affected by treatment (Fig. 4A, Table 2), and the body condition change of female juveniles was more positive in the more predictable treatment (Fig. 5A). Third, in 2013 adults grew less in the more predictable treatment from September to spring, suggesting that inter-age class competition may have been higher. Nevertheless, no treatment effects existed on yearlings from September to spring, and the body condition change of female juveniles was positive in the more predictable treatment (Fig. 5A), i.e. opposite to the direction predicted by the suggested inter-age class competition. Fourth, there was a consistent negative effect of less predictable precipitation on yearling growth during summer and autumn (Fig. 4A), that let to reduced spring body size (Fig. 4B), suggesting that inter-class competition among yearlings and juveniles may have been highest in the less predictable treatment. Nevertheless, only the change in body condition of female juveniles was in line with inter-age class competition, while the change in body condition of juvenile males was positive in less predictable conditions and negative in more predictable conditions (Fig. 5A). Most importantly, while treatment effects existed on growth of yearlings, no treatment effects existed on growth of juveniles (Table 2), a trait known to rapidly respond to inter-age class competition, at least in *Z. vivipara*^5^. Fifth, if adult growth between September and spring would reflect a pure life-history strategy, that responded to previous but not to the prevailing environmental conditions, one would predict that reduced growth of individuals exposed to more predictable conditions would lead to reduced competition among age-classes. Nevertheless, no significant treatment effects existed on yearling traits (Fig. 4A) and only body condition of juvenile females would have been positively affected (Fig. 5A), but neither juvenile males, nor juvenile growth. Sixth, there was indeed only one effect that might be in line with differences in inter-age class competition due to environmental predictability: adults and yearlings were growing less in the less predictable compared to the more predictable treatment from August to September 2013 (estimate ± SE for adults: -0.029 mm/day ± SE, yearlings: -0.025 ± 0.001 SE). However, the magnitude of the effect was similar (see estimates) and no effects existed on juvenile growth (Table 2). Consequently, it is unlikely that the differential treatment effects observed in the different age classes are the result of treatment-induced differences in inter-age class competition (prediction 3).

**Appendix S3**. Potential causes of differences between age-classes in the sensitivity to precipitation predictability.

Differences among treatment levels and age classes in the sensitivity to precipitation predictability may have arisen due to differences in thermal inertia and heating up by means of thermoregulation. If precipitation is falling, common lizards hide and are thus not able to thermoregulate, and differences in thermoregulation directly feed back into growth and timing of egg laying^7^. Moreover, thermoregulatory capacity is known to depend on body size^8^ and on coloration^9^. Larger animals (adults) have higher thermal inertia and can keep heat for longer^8^. Thus, if the precipitation event is not too long adults may keep heat, while smaller individuals may rapidly cool down, but note, smaller individuals also heat up more rapidly than larger animals^10^. Moreover, darker coloration (e.g. black colour exhibited by juveniles), leads to faster heating^9^. Only in 2013, the year with the coolest autumn temperatures (Fig. S5), less predictable precipitation let to reduced adult growth during autumn (Fig. 2), which is in line with predictability treatment-induced less regular thermoregulatory possibilities. Moreover, no significant treatment differences on adult growth existed in autumn 2012 and 2014 (Fig. 2), which is in line with higher autumn temperatures in 2012 and 2014 (Fig. S5) leading to a minor effect of less regular precipitation. In yearlings, growth was significantly lower in summer and autumn in the less predictable treatment, suggesting that given their small body size, they may have suffered more from less predictable thermoregulation due to reduced thermal inertia and the grey-brown dorsal coloration that may not have allowed to heat up as fast as juveniles. In contrast, growth of the black juveniles was not affected in any year, which is in line with fast heating-up of small-sized lizards and the thermoregulatory advantages of black coloration^9,10^. Similar to the growth patterns, treatment effects on laying date may as well have arisen due to treatment-induced differences in thermoregulation. April temperatures in 2015 were intermediate to those in 2013 and 2014 (Fig. S1), and significant treatment effects on laying date only existed in 2015. While April temperatures in 2013 may have been too low for reproductive activity (even after an increase in body temperature due to thermoregulation), those in 2014 were already high and thus slight treatment effects on thermoregulation may not have been important enough to affect growth, leading to no significant treatment effects. In contrast, in presence of intermediate temperatures, thermoregulating more regularly may have been an advantage allowing to lay the eggs 5 days ± 1.42 SE earlier. Our results are in line with prediction (4), that age-dependent effects of environmental predictability reflect differential sensitivities^11^, rather than inter-age class competition, and they suggest that the observed effects may have arisen through differences in thermal inertia and heating up due to thermoregulatory activity.

Table S1. Number of adults, yearlings, and juveniles released per enclosure and year.

|  | **Adults** | | | **Yearlings** | | | **Juveniles** | | | **Total** |
| --- | --- | --- | --- | --- | --- | --- | --- | --- | --- | --- |
|  | *Male* | *Female* | *Total* | *Male* | *Female* | *Total* | *Male* | *Female* | *Total* |  |
| 2012 | 7 | 13 | 20 | 6 | 6 | 12 | 18.75 ± 1.06 | 9.67 ± 1.30 | 28.42 ± 0.79 | 60.4 ± 0.79 |
| 2013 | 10 | 17 | 27 | 2 | 2 | 4 | 14.83 ± 1.9 | 5.67 ± 1.67 | 20.5 ± 1.57 | 51.5 ± 1.57 |
| 2014 | 9 | 18 | 27 | 2 | 2 | 4 | 15.25 ± 2.14 | 6.83 ± 2.12 | 22.08 ± 1.44 | 53.08 ± 1.44 |

Table S2. Minimum adequate models and test statistics for growth and change in body condition (BC) per age class. Significant factors and/or interactions are plotted in bold.

|  |  | **Adults** | | | | **Yearlings** | | | | **Juveniles** | | | |
| --- | --- | --- | --- | --- | --- | --- | --- | --- | --- | --- | --- | --- | --- |
|  |  | **Growth** | | **Change in BC** | | **Growth** | | **Change in BC** | | **Growth** | | **Change in BC** | |
| *Parameter* | *df* | *χ2* | *P* | *χ2* | *P* | *χ2* | *P* | *χ2* | *P* | *χ2* | *P* | *χ2* | *P* |
| Treatment | 1 | 1.309 | 0.253 | 0.006 | 0.940 | 1.925 | 0.165 | - | - | - | - | - | - |
| **Sex** | 1 | 4.871 | 0.027 | **12.223** | **<0.001** | **40.906** | **<0.001** | **19.553** | **<0.001** | - | - | - | - |
| **Year** | 2 | 12.919 | 0.002 | **39.837** | **<0.001** | 0.885 | 0.643 | **6.943** | **0.031** | - | - | **10.053** | **0.040** |
| **Period** | 2 | 58.112 | <0.001 | **11.110** | **0.004** | **596.062** | **<0.001** | **14.232** | **0.001** | - | - | **8.954** | **0.011** |
| Treatment x Sex | 1 | - | - | 2.001 | 0.157 | - | - | - | - | - | - | - | - |
| Treatment x Year | 2 | 1.480 | 0.477 | 3.738 | 0.154 | - | - | - | - | - | - | - | - |
| **Sex x Year** | 2 | - | - | 2.945 | 0.229 | **14.337** | **0.001** | 0.055 | 0.973 | - | - | - | - |
| **Treatment x Period** | 2 | 1.717 | 0.424 | 1.817 | 0.403 | **19.591** | **<0.001** | - | - | - | - | - | - |
| **Sex x Period** | 2 | **13.764** | **0.001** | **6.559** | **0.038** | **16.324** | **<0.001** | **6.205** | **0.045** | - | - | - | - |
| **Year x Period** | 4 | **253.954** | **<0.001** | **371.035** | **<0.001** | **38.801** | **<0.001** | **112.938** | **<0.001** | - | - | **10.053** | **0.040** |
| Treatment x Sex x Year | 2 | - | - | 1.776 | 0.412 | - | - | - | - | - | - | - | - |
| Treatment x Sex x Period | 2 | - | - | 0.467 | 0.792 | - | - | - | - | - | - | - | - |
| **Treatment x Year x Period** | 4 | **21.448** | **<0.001** | 6.833 | 0.145 | - | - | - | - | - | - | - | - |
| **Sex x Year x Period** | 4 | - | - | 6.055 | 0.195 | - | - | **14.524** | **0.006** | - | - | - | - |
| **Treatment x Sex x Year x Period** | 4 | - | - | **13.917** | **0.008** | - | - | - | - | - | - | - | - |

Table S3. Minimum adequate models and test statistics for final SVL and final body condition (BC) per age class. Significant factors and/or interactions are shown in bold.

|  |  | **Adults** | | | | **Yearlings** | | | | **Juveniles** | | | |
| --- | --- | --- | --- | --- | --- | --- | --- | --- | --- | --- | --- | --- | --- |
|  |  | **Final SVL** | | **Final BC** | | **Final SVL** | | **Final BC** | | **Final SVL** | | **Final BC** | |
|  | *df* | *χ2* | *P* | *χ2* | *P* | *χ2* | *P* | *χ2* | *P* | *χ2* | *P* | *χ2* | *P* |
| Treatment | 1 | - | - | - | - | **4.799** | **0.028** | - | - | - | - | 1.012 | 0.315 |
| Sex | 1 | **189.922** | **<0.001** | **406.399** | **<0.001** | **24.923** | **<0.001** | **139.553** | **<0.001** | - | - | 0.940 | 0.332 |
| Year | 2 | **9.455** | **0.009** | **12.952** | **0.002** | **14.545** | **0.001** | **12.110** | **0.002** | **36.531** | **<0.001** | - | - |
| Treatment x Sex | 1 | - | - | - | - | - | - | - | - | - | - | **6.679** | **0.010** |

Table S4. Minimum adequate models and test statistics for survival per age class. Significant factors and/or interactions are shown in bold.

|  |  | **Adults** | | **Yearlings** | | **Juveniles** | |
| --- | --- | --- | --- | --- | --- | --- | --- |
| *Parameters* | *df* | *χ2* | *P* | *χ2* | *P* | *χ2* | *P* |
| Treatment | 1 | - | - | - | - | - | - |
| Sex | 1 | 0.141 | 0.708 | - | - | - | - |
| **Year** | 2 | **13.500** | **0.001** | - | - | **2.6E6** | **<0.001** |


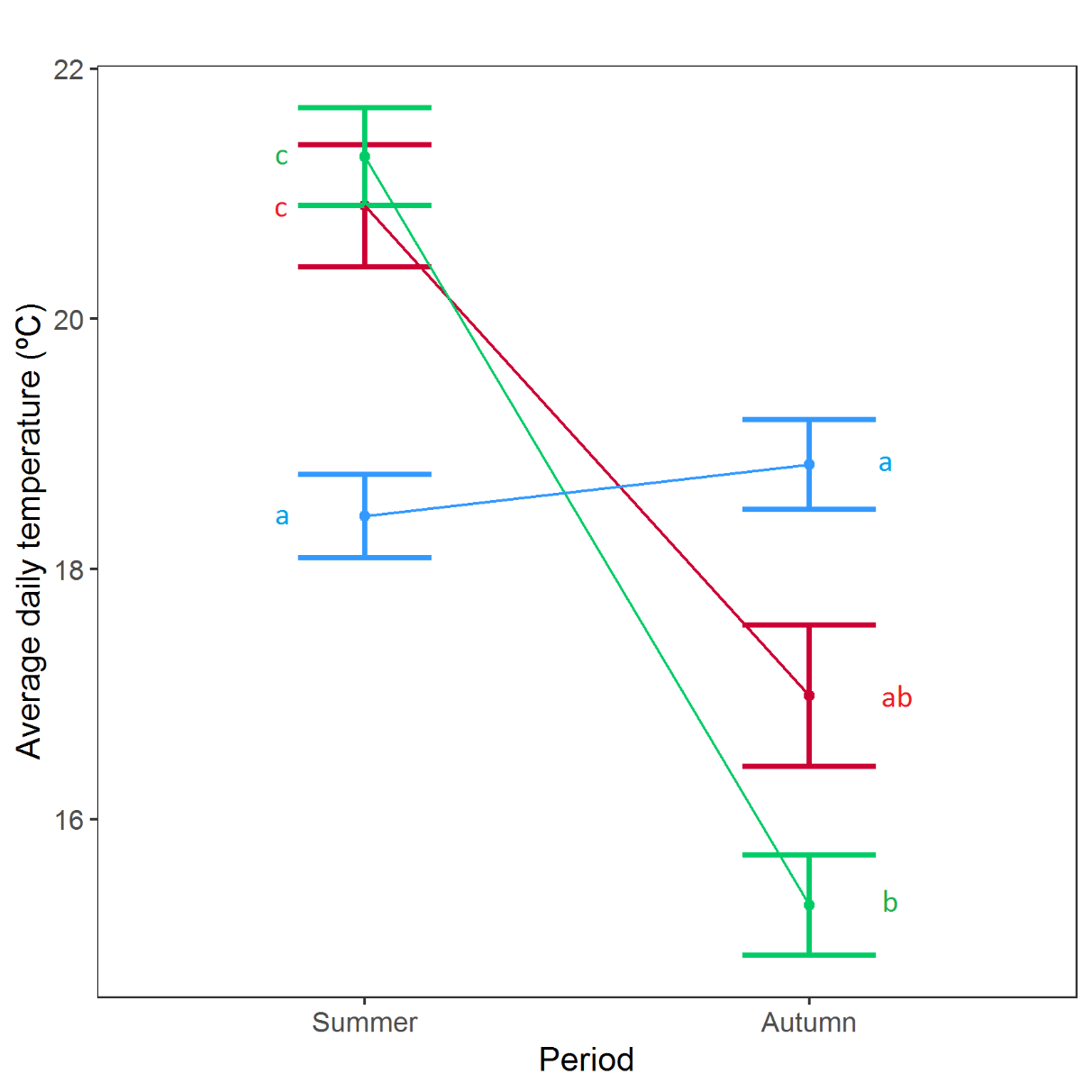


Figure S1. Differences between growth periods, summer (release - August) and autumn (August - September), and years in average daily temperature (°C). Red: 2012, green: 2013, blue: 2014. Given are means ± standard errors. Significant pairwise post-hoc comparisons are indicated with different letters.


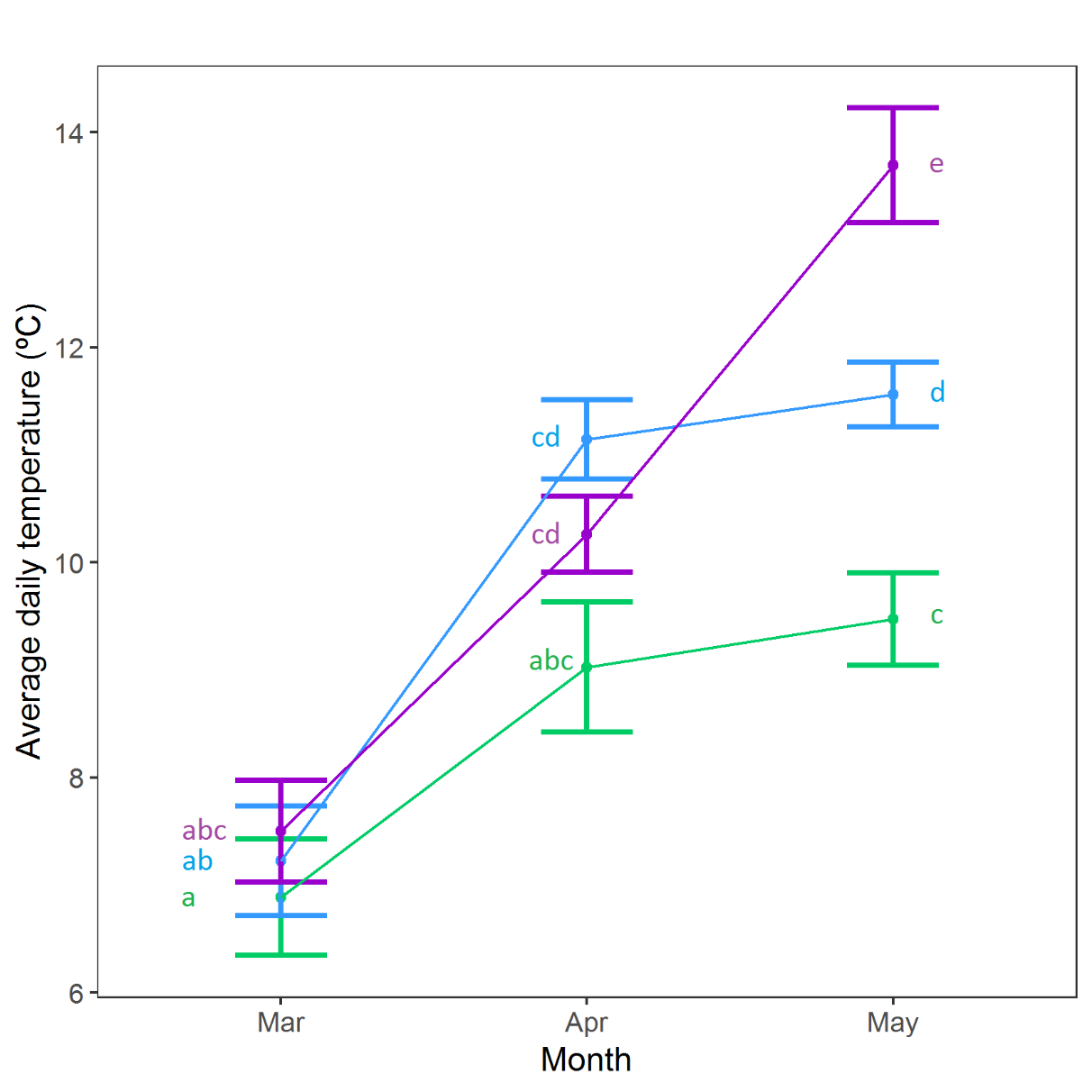


Figure S2. Differences between month and years in average daily temperatures (°C) during spring (March-May). Green: 2013, blue: 2014, violet: 2015. Given are means ± standard errors. Note, individuals of the 2012 experiment were exposed to spring temperatures in 2013, those of the 2013 to spring temperatures in 2014, etc. Significant pairwise post hoc comparisons are indicated with different letters.


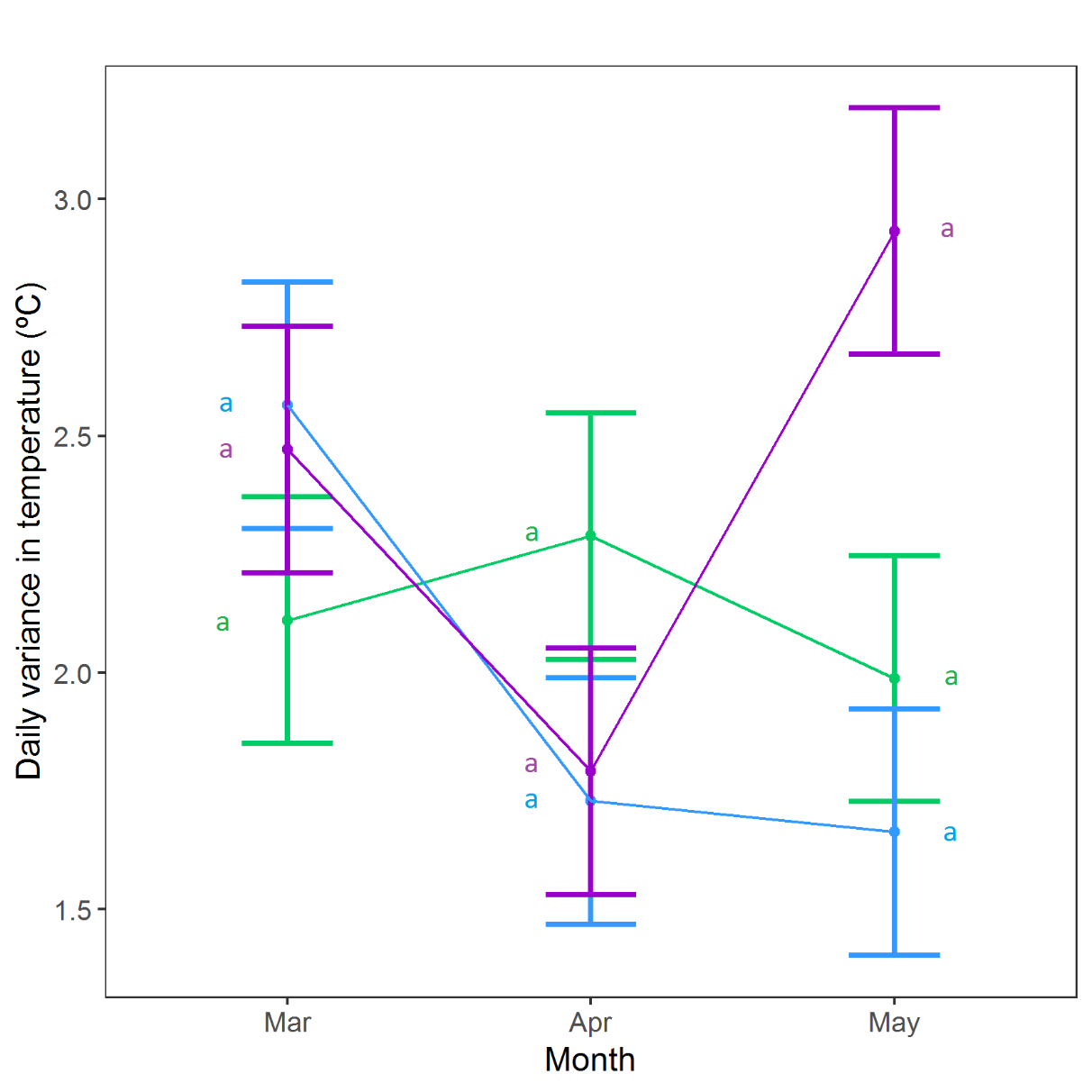


Figure S3. Differences between month and years in daily temperature variance (°C) during spring (March-May). Green: 2013, blue: 2014, violet: 2015. Given are variance means ± standard errors. Note, individuals of the 2012 experiment were exposed to spring temperatures in 2013, those of the 2013 to spring temperatures in 2014, etc. Significant pairwise post hoc comparisons are indicated with different letters.

*
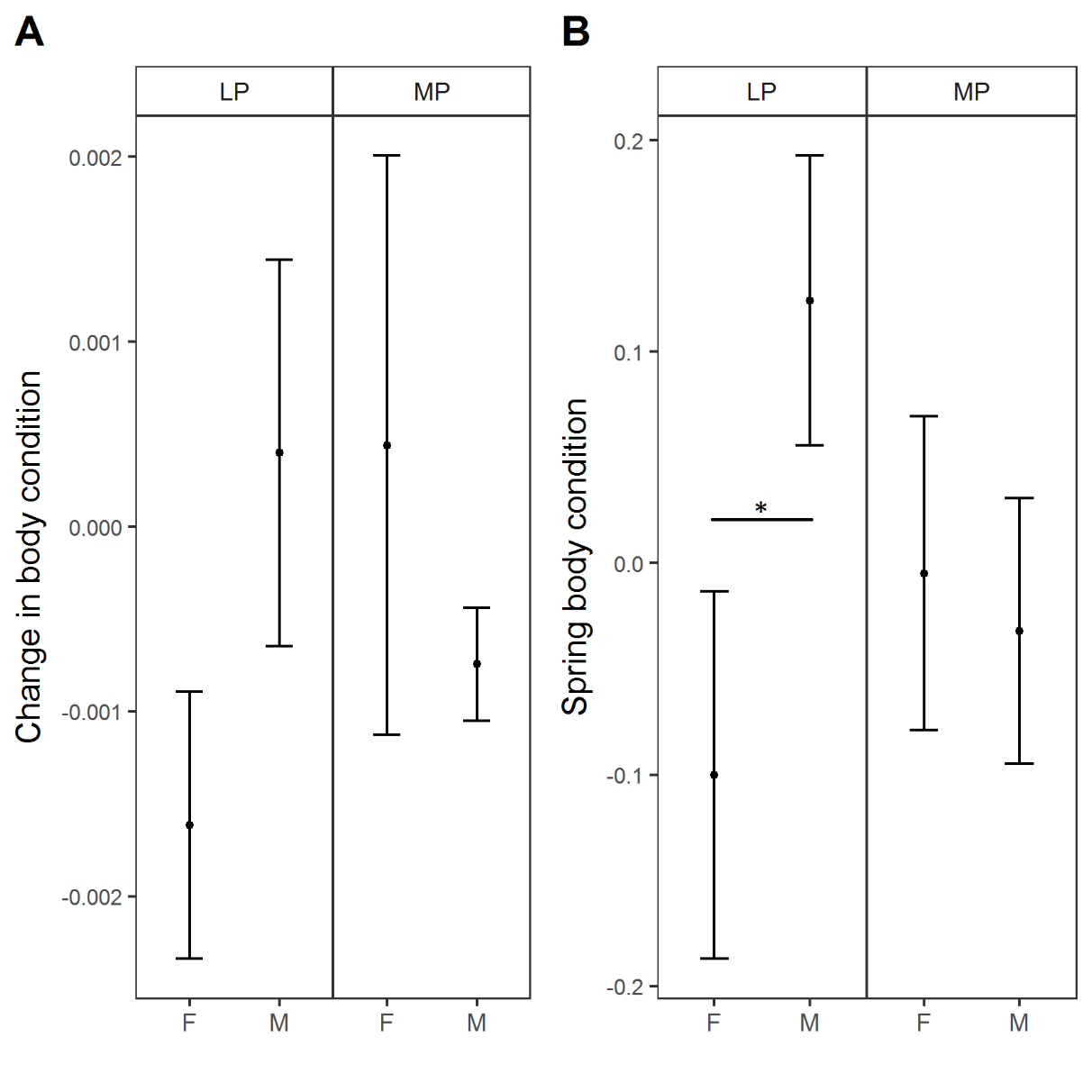
*

Figure S4. Treatment effects on the change in body condition (A) and on spring body condition (B) of juveniles. Shown are predicted means ± se per treatment and sex (LP: less predictable; MP: more predictable; sex: M: Males; F: Females). Horizontal lines indicate significant post-hoc contrasts: * *P* < 0.05; ** *P* < 0.01; *** *P* < 0.001.


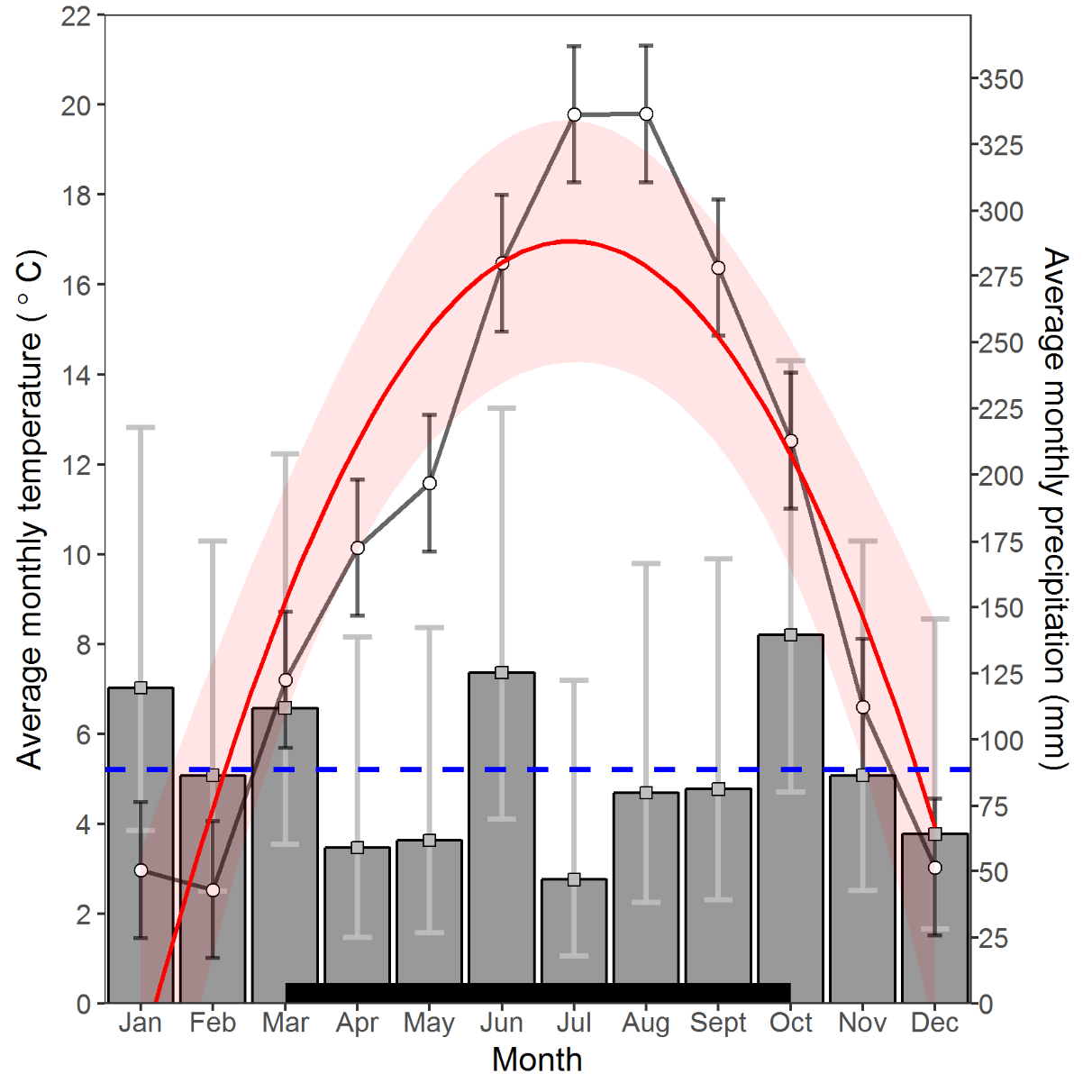


Figure S5. Monthly natural precipitation (in mm) and average monthly temperature (in °C) measured at the experimental field site over the duration of the experiment (2012 - 2015). Shown are average ± standard errors, for precipitation (grey bars) and temperature (white dots). The red line corresponds to model predictions of average monthly temperature in °C and the blue dashed line corresponds to the predicted average monthly precipitation. The black bar corresponds to the months during which lizards are active.

**Supporting information references**

1. Garland, J. & Bradley, E. Prediction in projection. *Chaos* **25**, 123108 (2015).

2. Riedl, M., Müller, A. & Wessel, N. Practical considerations of permutation entropy: A tutorial review. *Eur. Phys. J. Spec. Top.* **222**, 249–262 (2013).

3. Bandt, C. & Pompe, B. Permutation Entropy: A Natural Complexity Measure for Time Series. *Phys. Rev. Lett.* **88**, 4 (2002).

4. Pennekamp, F. *et al.* The intrinsic predictability of ecological time series and its potential to guide forecasting. *Ecol. Monogr.* **89**, e01359 (2019).

5. San-Jose, L. M., Peñalver-Alcázar, M., Huyghe, K., Breedveld, M. C. & Fitze, P. S. Inter-class competition in stage-structured populations: effects of adult density on life-history traits of adult and juvenile common lizards. *Oecologia* **182**, 1063–1074 (2016).

6. Heulin, B. Estival diet and use of trophic resources in three populations of Lacerta vivipara. *Oecologica Oecologia Gen.* **7**, 135–150 (1986).

7. Huey, R. B. & Slatkin, M. Cost and Benefits of Lizard Thermoregulation. *Q. Rev. Biol.* **51**, 363–384 (1976).

8. Christian, K. A., Tracy, C. R. & Tracy, C. R. Evaluating Thermoregulation in Reptiles: An Appropriate Null Model. *Am. Nat.* **168**, 421–430 (2006).

9. Clusella-Trullas, S., Van Wyk, J. H. & Spotila, J. R. Thermal benefits of melanism in cordylid lizards: A theoretical and field test. *Ecology* **90**, 2297–2312 (2009).

10. Herczeg, G., Török, J., Korsós, Z. & Herczeg, G. Size-dependent heating rates determine the spatial and temporal distribution of small-bodied lizards. *Amphibia-Reptilia* **28**, 347–356 (2007).

11. Rozen-Rechels, D. *et al.* Water restriction in viviparous lizards causes transgenerational effects on behavioral anxiety and immediate effects on exploration behavior. *Behav. Ecol. Sociobiol.* **72**, 23 (2018).
